# Ki-67 and outcome in clinically localised prostate cancer: analysis of conservatively treated prostate cancer patients from the Trans-Atlantic Prostate Group study

**DOI:** 10.1038/sj.bjc.6604951

**Published:** 2009-03-17

**Authors:** D M Berney, A Gopalan, S Kudahetti, G Fisher, L Ambroisine, C S Foster, V Reuter, J Eastham, H Moller, M W Kattan, W Gerald, C Cooper, P Scardino, J Cuzick

**Affiliations:** 1 The Orchid Tissue Laboratory, Centre for Molecular Oncology, Barts and The London School of Medicine and Dentistry, London, UK; 2 Department of Pathology, Memorial Sloan-Kettering Cancer Center, New York, NY, USA; 3 Cancer Research UK Centre for Epidemiology, Mathematics and Statistics, Wolfson Institute of Preventive Medicine, St Bartholomew's Medical School, Queen Mary, University of London, London, UK; 4 Department of Cellular Pathology and Molecular Genetics, Liverpool University Hospital, Liverpool, UK; 5 Department of Urology, Memorial Sloan-Kettering Cancer Center, New York, NY, USA; 6 King's College London, Thames Cancer Registry, London, UK; 7 Department of Quantitative Health Sciences, Cleveland Clinic, Cleveland, OH, USA; 8 Institute of Cancer, The Royal Marsden Hospital, Sutton, UK

**Keywords:** prostate cancer, Ki-67, watchful waiting, active surveillance, biomarker

## Abstract

Treatment decisions after diagnosis of clinically localised prostate cancer are difficult due to variability in tumour behaviour. We therefore examined one of the most promising biomarkers in prostate cancer, Ki-67, in a cohort of 808 patients diagnosed with prostate cancer between 1990 and 1996 and treated conservatively. Ki-67 expression was assessed immunohistochemically, in two laboratories, by two different scoring methods and the results compared with cancer-specific and overall survival. The power of the biomarker was compared with Gleason score and initial serum prostate-specific antigen (PSA). Both methods showed that Ki-67 provided additional prognostic information beyond that available from Gleason score and PSA: for the semi-quantitative method, Δ*χ*^2^ (1 d.f.)=24.6 (*P*<0.0001), overall survival *χ*^2^=20.5 (*P*<0.0001), and for the quantitative method, Δ*χ*^2^ (1 d.f.)=15.1 (*P*=0.0001), overall survival *χ*^2^=10.85 (*P*=0.001). Ki-67 is a powerful biomarker in localised prostate cancer and adds to a model predicting the need for radical or conservative therapy. As it is already in widespread use in routine pathology, it is confirmed as the most promising biomarker to be applied into routine practice.

In spite of extensive research, there are very few molecular markers that have been translated into routine use in clinical oncology. Any new biomarker must give further predictive ability beyond that available by using current known, easily measured parameters, and the data must differentiate patients into groups for which different treatment options can be selected. Biomarker measurement for purely prognostic information has limited utility unless it indicates different treatment options.

In no area of oncology are new biomarkers more urgently required than in prostate cancer. The most appropriate treatment for clinically localised prostate cancer is still unknown. Treatment options vary from radical surgery (Bott *et al*, 2005) or radiotherapy (Parker and Dearnaley, 2003) to hormonal treatment alone, or simply active surveillance and watchful waiting (Albertsen *et al*, 2005; Parker *et al*, 2006). Patients must choose, with little scientific rationale, between these wildly contrasting treatments. Screening for prostate cancer in the United States has caused an almost doubling incidence, whereas mortality has remained relatively stable (McDavid *et al*, 2004). Radical treatments have the advantage of a high cure rate. However, this comes with the potential of serious long-term morbidity from sexual dysfunction or incontinence. Many tumours do not need such radical intervention, given that the majority of men die ‘with’ their prostate cancer rather than ‘from’ it. A more conservative approach with regular monitoring of clinical parameters, such as serum prostate-specific antigen (PSA) and possibly rebiopsy and intervention if necessary, has become a viable treatment option (Parker *et al*, 2006), but 20–30% of men on active surveillance go on to receive radical treatments, and to select the appropriate patient for this management strategy remains a challenge.

Although Gleason score and serum PSA have been shown to be strong predictors of prognosis in early prostate cancer, much remains to be done to improve predictions. There remains a middle range of prostatic tumours (Gleason score <7 and low PSA), where it is not possible with current methods to predict recurrence. The search for biomarkers that can add to the knowledge provided by the known markers is therefore of high importance in future as a guide to therapy.

The Ki-67 protein is well known and widely used to assess the tumour proliferation rate. It is one of the several cell-cycle-regulating proteins, which can be demonstrated by immunohistochemistry (Gerdes *et al*, 1983). It is a DNA-binding protein that is expressed in all phases of cell cycle but undetectable in resting cells (Gerdes *et al*, 1991) though strangely its function is yet to be clearly elucidated. Numerous studies have shown Ki-67 to be a prognostic marker in prostate cancer treated by radical prostatectomy, but none in a large conservatively treated cohort of prostate cancers with long-term follow-up in which clear relative risk values can be calculated. Our hypothesis was that measurement of Ki-67 in such a well-defined cohort could provide prognostic information that supplemented the currently known indicators of prognosis in prostate cancer.

## Materials and methods

The Trans-Atlantic Prostate Group (TAPG) of clinicians and scientists from the United States and the United Kingdom have assembled the largest cohort of prostate cancers treated by conservative means with both initial serum PSA levels and centralised Gleason scoring. The detailed methods of cohort assembly have been described in an earlier paper (Cuzick *et al*, 2006). In short, men were included in this study if they were under 76 years of age at diagnosis and had clinically localised prostate cancer diagnosed between January 1990 and December 1996. Patients who had a radical prostatectomy or radiation therapy within 6 months of diagnosis, or clear evidence of metastatic disease (by bone scan, X-ray, CT scan, MRI, bone biopsy, lymph node biopsy or pelvic lymph node dissection) or clinical indications of metastatic disease (including pathologic fracture, soft tissue metastasis, spinal compression or bone pain) at or within 6 months of diagnosis, were excluded. Eligibility was established by review of patient records by registry data-collection officers and trained medical staff. Clinical staging was centrally reviewed. All patients had centralised Gleason grading by a panel of genitourinary pathologists and had initial diagnostic serum PSA available. Blocks from the trans-urethral resection specimens, which were available, were identified and the corresponding haematoxylin and eosin sections marked for cancerous areas. These were microarrayed in a series of 24 blocks using 0.6 mm cylinders of tissue. Four cores were taken from different areas of tumour to account for tumour heterogeneity in each case, and areas of adjacent normal tissue were also sampled.

The sections were immunostained for Ki-67 using DAKO (MIBI, Carpinteria, CA, USA), with an overnight incubation at 4°C, 1 : 200 dilution and pretreatment with citric acid, pH 6.0. Immunostaining for p63 and CK5/6 were also performed to ensure that only cancerous areas were identified when the cores were examined. These are immunohistochemical markers of prostatic basal cells and are absent in areas of prostatic carcinoma. Normal tonsil was used as a positive control where a high proliferation is seen in germinal centres.

The samples were all examined in a semi-quantitative manner (DB/SK) giving each tumour core an estimated percentage of positive cells, in a manner similar to that used in routine pathology departments for the assessment of proliferation index in other organs. A more formalised quantitative approach was also undertaken (AG/WG). Slides were scanned at × 4 for any nuclear staining. A magnification of × 10 was used to verify that the staining was in carcinoma. The total number of carcinoma cells in a given core was estimated at × 10 and estimated as low, intermediate or high cellularity corresponding to ⩽99, 100–299 and ⩾300 cells (number of cells was recorded as multiples of 25). The number of Ki-67-positive cells was then individually counted at × 10 magnification. A percentage was derived using the number of Ki-67-positive cells as the numerator and the total number of malignant cells (invasive carcinoma) as the denominator. Estimation of Ki-67 positivity was not carried out when thermal/crush artefact precluded evaluation of individual cells. In common with other microarray studies, the maximal staining from the sampled cores for each case was taken. For each patient, the maximum value of Ki-67 % staining in all cancerous cores was calculated and used for analysis. The results were analysed by dichotomous, grouped and continuous variables and compared with overall and prostate cancer-specific survival. The effect of Ki-67 was evaluated by multivariate comparisons in a model including Gleason score, serum PSA and extent of disease and with ETS (ERG–ETV1 fusion gene rearrangement) status.

## Results

**Figure 1:**
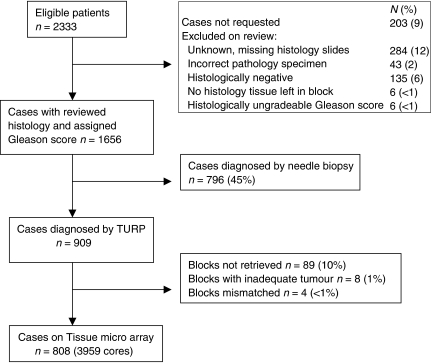
Tissue microarray cohort derivation.

A total of 808 eligible cases were included on the tissue microarray. Their derivation is described in Figure 1. Ki-67 staining results were analysed for both the semi-quantitative and quantitative methods.

Analysis included 1798 Ki-67-stained cancerous cores, corresponding to 693 cases. Characteristics of the cohort are given in Table 1.

**Figure 2:**
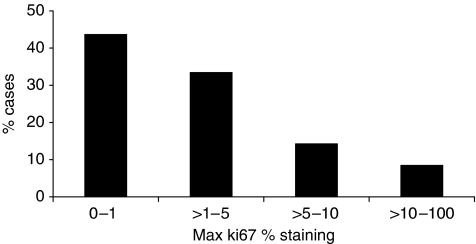
Distribution of Ki-67 % staining in cases, *n*=693.

The mean Ki-67 score was 5.42%, with a standard deviation of 6.98% and a range of 0–60% (Figure 2).

The prognostic value of Ki-67, as a biomarker for prostate cancer-specific and overall survival, was assessed as a continuous variable, as a grouped variable with limits 0, 1, 5, 10, >10–100 and as a dichotomous variable with a cutoff at ⩽5%.

**Figure 3:**
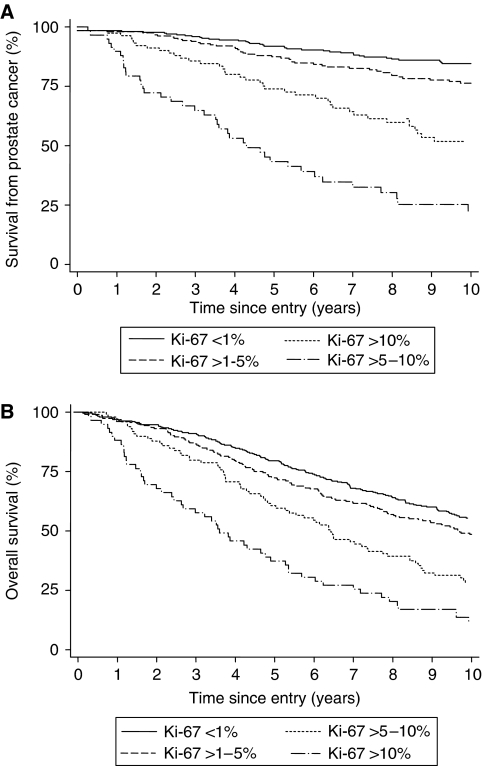
(**A** and **B**) Prostate cancer survival and overall survival as predicted by four groups of Ki-67 score.

In univariate analysis, the most useful variable was Gleason score (*χ*^2^(trend)=164), followed by extent of the disease, Ki-67, baseline PSA, clinical stage and ETS status. The discriminating power of age (*χ*^2^(trend)=4) and year of diagnosis (*χ*^2^(trend)=6.2) was low. Results were similar to those reported for the total cohort. Gleason score was the most informative variable (*χ*^2^(trend)=164), but the extent of disease appeared a stronger variable than baseline PSA (*χ*^2^(trend)=116 and 87, respectively). The level of Ki-67 staining was a significant prognostic factor for cause-specific survival (HR=1.09, 95% CI=1.07–1.10, *P*<0.001) and overall survival (HR=1.06, 95% CI=1.05–1.07, *P*<0.001). Significance was maintained whether the results were analysed as a continuous variable or as groups (Table 1; Figure 3A and B). A more fully quantitative method assessed by counting cells (AG and WG) did not improve the predictive value (Table 1).

The three strongest variables, Gleason score, baseline PSA and extent of disease, were included in a multivariate model together with Ki-67. Results for all four variables were available for 685 cases (99%). The level of Ki-67 staining remained an independent prognostic factor of cause-specific survival (Δ*χ*^2^ (1 d.f.)=24.6, *P*<0.0001) and overall survival (*χ*^2^=20.5, *P*<0.0001) (Table 2). Again, the use of a more fully quantitative method was not better than the semi-quantitative method reported in this table (details not shown).

ERG–ETV1 rearrangement status has been shown to be an important predictor for prostate cancer cause-specific survival. In univariate analysis, the presence or absence of ETS was significant for cause-specific survival (HR=2.38, 95% CI=1.7–3.3, *P*<0.0001). However, ETS status was not significant independently of Gleason score, PSA and extent of disease in multivariate analysis (Δ*χ*^2^ (1 d.f.)=0.64, *P*=0.42).

We evaluated Ki-67 in both ETS-positive and ETS-negative subsets and found similar predictive values in both subgroups (HR 1.32, 95% CI 1.0–1.7, *vs* 1.61, 95% CI 1.2–2.2, respectively), and the test for heterogeneity was not significant (*P*=0.5).

In subgroups based on Gleason score, the independent prognostic significance of Ki-67 for cause-specific survival was maintained in men with Gleason score >7 (*P*<0.0001), but not in men with Gleason score ⩽7, and similarly for overall survival, in men with Gleason score >7 (*P*=0.0003) (Table 2).

In risk groups using Gleason score and baseline PSA, identified earlier as high (prostate cancer mortality at 10 years >30%), intermediate and low risk (prostate cancer mortality at 10 years <10%), Ki-67 was a significant independent prognostic factor for cause-specific survival in men at high risk (*P*=0.0001) or intermediate risk (*P*=0.049), but not among those at low risk of dying from prostate cancer, possibly because of the very few cases of men with a low Gleason score and high Ki-67 (*P*=0.99).

## Discussion

Thousands of patients undergo radical treatment for prostate cancer every year in Europe and the United States. Many of these patients will have curative and beneficial treatment. However, a significant number of these patients have treatment unnecessarily as their tumours will not progress before death from another cause. They may suffer unnecessary morbidity from their surgery.

This was recognised in the recently published National Institute of Clinical Excellence (NICE) prostate cancer guidelines in the United Kingdom, which states under its key research recommendations that ‘Further research is required into the identification of prognostic indicators in order to differentiate effectively between men who may die with prostate cancer and those who might die from prostate cancer. The greatest uncertainties in managing prostate cancer are around the identification of which cancers are of clinical significance and over the choice of radical treatment, and in which settings they are appropriate. With the diagnosis of prostate cancer being made more frequently in asymptomatic men, it is of growing importance to know which of these men are likely to benefit from aggressive treatment.’ (http://www.nice.org.uk/nicemedia/pdf/CG58FullGuideline.pdf, 2008).

Numerous studies have shown Ki-67 to be a prognostic indicator in various stages of prostate cancer. However, to our knowledge, there are no studies conducted on a large conservatively treated cohort with outcome data, where decisions on future treatment could be indicated, and a very recent paper (Laurila *et al*, 2009) suggested that further studies are needed to show the final outcome of proliferative activity in patients with watchful waiting.

Since the initial studies showing the utility and feasibility of immunochemistry on paraffin-embedded formalin-fixed urological material (Fontana *et al*, 1987), Ki-67 was shown to be an interesting marker in prostate cancer at an early stage (Oomens *et al*, 1991; McLoughlin *et al*, 1993). It has been investigated on TURP material (Feneley *et al*, 1996), biopsy material (Szende *et al*, 1999; Cowen *et al*, 2002; Ojea *et al*, 2004) and radical prostatectomy series (Bettencourt *et al*, 1996; Moul, 1999; Zudaire Bergera *et al*, 2000; Inoue *et al*, 2005) in large screening programmes (Laurila *et al*, 2009) and after radiation therapy (Li *et al*, 2004). Some of these show that Ki-67 is an independent predictor of outcome on multivariate analysis (Pollack *et al*, 2004). This is the largest series to date with marker analysis and one that utilises the currently used prognostic biomarkers of serum PSA and Gleason score. This is of great relevance as all of these cancers were treated conservatively at diagnosis, and therefore the Ki-67 score would have given further information on recurrence risk and enhanced the decision making process of radical *vs* conservative management. Our earlier papers on this cohort have shown the discriminatory value of revised Gleason score and serum PSA at predicting tumour behaviour (Cuzick *et al*, 2006; Kattan *et al*, 2007; Eastham *et al*, 2008). However, the natural history of 60% of the cohort, where the Gleason score was 6 or 7 and with moderate PSA rises, was in doubt.

Ki-67 has a number of advantages as a biomarker. First, it is widely used by academic and non-academic Histopathology departments, which have the ability to perform immunochemistry, and is used as an adjunct to diagnosis and management in a number of other diseases. It is easily verified in control tissue and it is very easy to measure in a semi-quantitative manner, similar to ‘quick scores’ performed for receptor status in breast cancers. This study shows that semi-quantitative methodologies may produce as much information as more time-consuming methods that may not be possible for the average laboratory. Immunochemistry has the advantage over other molecular techniques that it is easily applied to paraffin-embedded tissue and has a very low ‘failure’ rate, unlike fluorescence. The TAPG group has shown in an earlier paper that fluorescence *in situ* hybridisation for the detection of the TMPSS/ERG translocation also identifies aggressive prostate cancer (Attard *et al*, 2008). However, such assessments can be difficult with small amounts of formalin-fixed material, and the recent discovery of complex rearrangements within single glands raises problems with tumour heterogeneity (Clark *et al*, 2008). These techniques are not available in most hospitals, and need careful assessment. Tumour tissue from the prostate is very difficult to identify macroscopically, and therefore assessment in this manner remains by far the simplest technique by which tissue biomarker assessment is likely to become widespread in the assessment of prostatic malignancy. From a biological point of view, Ki-67 use as a proliferation marker is also an adjunct to existing methods. Although many grading systems for cancers include mitotic count in the calculation of grade (such as the Nottingham method for assessing grade in breast carcinoma), the Gleason grading system is purely pattern based. Therefore, this may be a reason why the estimation of proliferation index appears such a powerful independent predictor of tumour behaviour. The other cardinal advantage of this cohort is the length of follow-up and the use of both cause-specific and overall death outcomes. The natural history of prostate cancer means that such long-term assessments are essential for the validation of new biomarkers.

A number of limitations of the study have to be noted. First, this is a retrospective cohort study dealing with prostate cancer diagnosis, as it was in the 1990s rather than it is presently. Most patients would have presented symptomatically, and the vast increase in prostate cancer diagnosis in the past 20 years is undoubtedly due to an increase in PSA screening. However, in all countries, prostate cancer diagnosis continues to be unscientific in its basis. Screening is unproven and instituted in relatively few centres. Health-care standards in the United States vary from intense screening, with a high cancer detection of clinically insignificant tumours in centres of excellence, to more symptom-based treatments in the disadvantaged parts of the population.

In the United Kingdom, there has been an increase in requests for PSA testing, but many men are only tested after complaining of urinary symptoms. Thus, despite an increase in public health awareness of prostate cancer, the disease is detected by a variety of methods, and we have moved from one unscientifically proven model to another, which is equally unproven. Screening, although popular politically, is not evidence based in prostate cancer. Hardly one criterion for screening assessment is fulfilled by prostate cancer. The medical community is therefore left with offering patients a variety of drastically different treatments, with no evidence base.

However, the major difference between this study and current practice is that it is an assessment of TURP material only. As biopsy diagnosis of prostate cancer is far more common, especially in developed countries, it could be argued that this study does not represent modern detection methods. The original cohort comprised 55% TURP and 45% biopsy-diagnosed tumours. Interestingly, the method of diagnosis had no significant difference on outcome in our cohort (Cuzick *et al*, 2006), and Gleason profiles of both limbs were comparable. The assessment of Ki-67 on the biopsy material, which forms the second half of this cohort, is planned to further validate these data, as well as computer-based assessments of Ki-67 index, to attempt validation of systems that can be used in different laboratories.

Despite intense interest, no biomarker is currently routinely utilised for the assessment of the degree of aggressiveness of prostate cancer. We have shown that Ki-67 is an interesting candidate for the routine assessment of suitability for active surveillance treatments and that this study needs to be confirmed in biopsy material.

## Figures and Tables

**Table 1:** Univariate analysis of factors influencing death from prostate cancer in men with conservatively managed clinically localised prostate cancer: total cohort (*n*=693)

**Variable**	***N* (%)**	**Hazard ratio (95% CI)**
*Gleason score* a		
⩽5	30 (4)	0.34 (0–2.5)
6	306 (44)	1^b^
7	183 (26)	3.25 (2.1–5.1)
8	86 (12)	5.58 (3.4–9.1)
9 or 10	88 (13)	14.56 (9.3–22.7)
		*χ*^2^(trend)=164
*Serum PSA (ng* *ml*^−*1*^*)*		
⩽4	233 (34)	1^b^
>4–10	139 (20)	2.13 (1.2–3.7)
>10–25	148 (21)	4.31 (2.6–7.2)
>25–50	107 (15)	5.6 (3.4–9.4)
>50–100	66 (10)	8.77 (5.1–15)
		*χ*^2^(trend)=87
*Clinical stage*		
T1	189 (27)	1^b^
T2	160 (23)	2.87 (1.7–4.8)
T3	80 (12)	5.83 (3.4–9.9)
Unknown^c^	264 (38)	
		*χ*^2^(trend)=45
*Cancer in biopsy* (%)		
⩽6	166 (24)	0.77 (0.4–1.5)
>6–20	172 (25)	1^b^
>20–40	102 (15)	2.47 (1.4–4.5)
>40–75	103 (15)	4.04 (2.3–7)
>75–100	142 (20)	6.66 (4.1–10.9)
Unspecified^c^	8 (1)	
		*χ*^2^(trend)=116
*Ki-67* % *groups (semi-quantitative)*		
0–1	303 (44)	1^b^
>1–5	232 (33)	1.59 (1.1–2.4)
>5–10	99 (14)	3.60 (2.3–5.5)
>10–100	59 (9)	9.25 (6–14.2)
		*χ*^2^(trend)=97
*Ki-67*% *class (semi-quantitative)*		
0–5	535 (77)	1^b^
>5	158 (23)	4.17 (3.1–5.6)
		*χ*^2^(trend)=78
*Ki-67* % *groups (quantitative)*^d^		
0–1	72 (11)	1^b^
>1–5	240 (36)	1.08 (0.6–2.0)
>5–10	171 (25)	1.17 (0.6–2.3)
>10–100	188 (28)	3.52 (1.9–6.4)
		*χ*^2^(trend)=45
*Ki-67*% *class (quantitative)*^d^		
0–5	312 (46)	1^b^
>5	359 (54)	2.14 (1.6–2.9)
		*χ*^2^(trend)=24

CI=confidence interval; PSA=prostate-specific antigen.

a Score assigned during histopathological review.

b Reference category.

c These cases were excluded from the trend analysis.

d Quantitative method: total cohort (*n*=671).

**Table 2:** The added value of Ki-67 in a multivariate model for prostate cancer-specific survival and overall survival, based on gleason grade, baseline PSA, percentage cancer in biopsy and Ki-67: Ki-67 staining was measured semi-quantitatively: total cohort (*n*=685)

	**Prostate cancer survival**			**Overall survival**		
**Added variable**	**Deaths**	**Δ** *χ* **^2^ (1 d.f.)**	***P*-value**	**Deaths**	**Δ** *χ* **^2^ (1 d.f.)**	***P*-value**
Ki-67 % (continuous)	173	24.55	<0.0001	418	20.52	<0.0001
Ki-67 % (groups)	173	18.55	<0.0001	418	11.97	0.0005
Ki-67 % (⩽5, >5)	173	13.41	0.0003	418	10.42	0.0012
						
*Gleason*<*7*						
Ki-67 % (groups)	31	0.01	0.91	153	1.11	0.29
						
*Gleason*=*7*						
Ki-67 % (groups)	48	2.02	0.15	125	1.02	0.31
						
*Gleason*>*7*						
Ki-67 % (groups)	94	20.63	<0.0001	140	13.15	0.0003

PSA=prostate-specific antigen.
